# Unusual Van der Waals Magnetoresistance in Stacked Ferromagnetic Fe_3_GeTe_2_: The Role of Atomically Sharp Interfaces

**DOI:** 10.1002/advs.202508244

**Published:** 2025-09-12

**Authors:** Qian Chen, Junwen Sun, Jian Liang, Wei Jiang, Zhipeng Yu, Zhaocong Huang, Zhongming Zeng, Ya Zhai, Ke Xia, Xiangrong Wang

**Affiliations:** ^1^ Key Laboratory of Quantum Materials and Devices of Ministry of Education School of Physics Southeast University Nanjing 211189 China; ^2^ School of Science and Engineering Chinese University of Hong Kong Shenzhen Shenzhen 518172 China; ^3^ Department of Physics The Hong Kong University of Science and Technology Clear Water Bay Kowloon Hong Kong China; ^4^ Nanofabrication Facility Suzhou Institute of Nano‐Tech and Nano‐Bionics Chinese Academy of Sciences Suzhou Jiangsu 215123 China

**Keywords:** anisotropic magnetoresistance, atomically sharp interfaces, interfacial field, two‐vector theory, van der Waals ferromagnet

## Abstract

Interfaces can significantly influence the physical properties of systems, especially in 2D van der Waals (vdW) magnets, where atomically sharp interfaces (ASI) are intrinsic. However, the role of the ASI fields on magnetoresistance (MR) in vdW magnetic layers has largely been overlooked. Here, we investigate the angular dependence of MR in stacked ferromagnetic Fe_3_GeTe_2_ (FGT). Remarkably, the MR exhibits similar universal behaviors associated with unusual anisotropic magnetoresistance (UAMR), which has been widely observed in almost all magnet/non‐magnet bilayers at the nanometer scale, featuring distinct characteristics. Unlike the exponential decay of UAMR with thickness in nanometer‐thick bilayers, the UAMR of stacked FGT layers remains insensitive to thickness. The MR in the film plane displays dominant two‐fold oscillation, while high‐order oscillations of MR exceeding 1.4% are observed in the planes perpendicular to the film, nearly an order of magnitude larger than in‐plane anisotropic MR. The UAMR of the FGT films cannot be explained by the well‐known spin Hall MR theory based on spin/orbital current and charge current interconversion. Instead, it aligns with the predictions of the two‐vector MR theory, particularly its sum‐rule constraints. The results provide direct experimental evidence that the ASI field, rather than spin/charge current interconversion, governs the UAMR in vdW ferromagnets.

## Graphical abstract

Angular‐dependent magnetoresistance in stacked Fe_3_GeTe_2_ reveals high‐order UAMR that defies spin‐current‐based interpretations. Instead, it originates from internal electric fields at atomically sharp interfaces (ASI), in excellent agreement with two‐vector MR theory. These results highlight ASI fields as key drivers of magnetoelectric effects in vdW magnets.

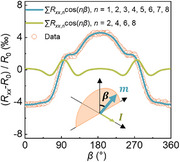

## 1 Introduction

Interfaces serve as fertile grounds for emergent physical phenomena, a concept championed by Kroemer, the 2000 Nobel Laureate in Physics, who highlighted the idea of interfaces as devices. The physical properties at interfaces, such as superconductivity, magnetism, and topological states,^[^ ^1^, ^2^, ^3^, ^4^ ^]^ can differ fundamentally from those in the bulk material. In the realm of spintronics, this is best exemplified by ferromagnet/nonmagnet (FM/NM) bilayers, where magnetoelectric transport is widely believed to be governed by mechanisms such as spin/charge current interconversion, scattering, and absorption.^[^ ^5^, ^6^, ^7^, ^8^ ^]^

As research progresses into 2D van der Waals (vdW) magnets—materials characterized by atomically sharp interfaces (ASI) between layers^[^ ^9^, ^10^ ^]^—it has become increasingly evident that these ASI fields may not only contribute but potentially dominate the underlying physics, particularly in magnetoelectric transport phenomena.

Despite their significance, intrinsic ASI fields can be exceptionally strong in layered vdW systems due to broken inversion symmetry and interlayer charge redistribution. However, the influence of these ASI fields on magnetotransport remains poorly understood. This gap is particularly apparent in the context of unusual anisotropic magnetoresistance (UAMR), which describes an angular‐dependent magnetoresistance (MR) effect extensively reported in FM/NM bilayers and often attributed to spin Hall magnetoresistance (SMR).^[^ ^11^, ^12^, ^13^, ^14^ ^]^ Within the SMR framework, changes in resistance are ascribed to spin accumulation and spin‐current absorption at interfaces as the magnetization rotates relative to the polarization of the spin current.^[^ ^11^, ^12^, ^13^, ^14^, ^15^, ^16^ ^]^ Interestingly, UAMR has also been observed in systems lacking strong spin–orbit coupling, as well as in magnetic single layers,^[^ ^17^, ^18^, ^19^, ^20^, ^21^, ^22^, ^23^, ^24^, ^25^, ^26^, ^27^ ^]^ revealing inconsistencies with spin‐current‐based explanations. In response, researchers have proposed various theoretical models, including Rashba‐Edelstein MR,^[^ ^17^, ^18^ ^]^ spin‐orbit MR,^[^ ^19^ ^]^ anomalous Hall MR,^[^ ^20^ ^]^ orbital Hall MR,^[^ ^21^ ^]^ orbital Rashba‐Edelstein MR,^[^ ^22^ ^]^ Hanle MR,^[^ ^23^ ^]^ and unidirectional MR.^[^ ^24^ ^]^ While each model addresses specific mechanisms, none has succeeded in providing a comprehensive and unified framework. The proliferation of models to explain a single widely observed phenomenon underscores a fundamental disconnect between experimental observations and theoretical predictions, highlighting the need for a more intrinsic mechanism that does not rely solely on spin‐current conversion.

A compelling alternative is the two‐vector MR theory,^[^ ^25^, ^26^, ^27^ ^]^ which attributes UAMR to the coupling between the magnetization and a built‐in interfacial electric field—naturally arising at ASIs. This theory effectively captures both longitudinal and transverse MR signatures and predicts quantitative constraints, such as sum rules linking harmonic components, that are independent of microscopic details. However, direct experimental validation in systems where ASIs are intrinsic, rather than artificially engineered, has remained elusive.

In this study, we investigate stacked Fe_3_GeTe_2_ (FGT) multilayers,^[^ ^28^, ^29^, ^30^ ^]^ a model vdW ferromagnet system characterized by a periodic array of atomically sharp interfaces and inherently weak crystalline anisotropy.^[^ ^31^, ^32^ ^]^ These features render FGT an ideal platform to isolate and probe the effects of ASI fields on UAMR. Our findings reveal that UAMR in stacked FGT films not only greatly exceeds conventional anisotropic MR but also displays high‐order angular harmonics—up to the 8th order—in both in‐plane and out‐of‐plane configurations. The magnitude of out‐of‐plane UAMR exceeds 1.4%, nearly an order of magnitude greater than its in‐plane counterpart. These results are inconsistent with SMR and related spin‐current‐based models but align both qualitatively and quantitatively with the two‐vector theory, including its harmonic sum‐rule constraints. Our results provide direct experimental evidence that ASI fields—not spin/charge current interconversion—govern the UAMR in vdW ferromagnets. Beyond confirming the two‐vector MR theory, this work positions ASI fields as a critical control knob for tuning magnetoelectric transport in low‐dimensional systems, offering a new framework for interface‐engineered spintronic functionalities.

## 2 Results and Discussion

**Figure 1:**
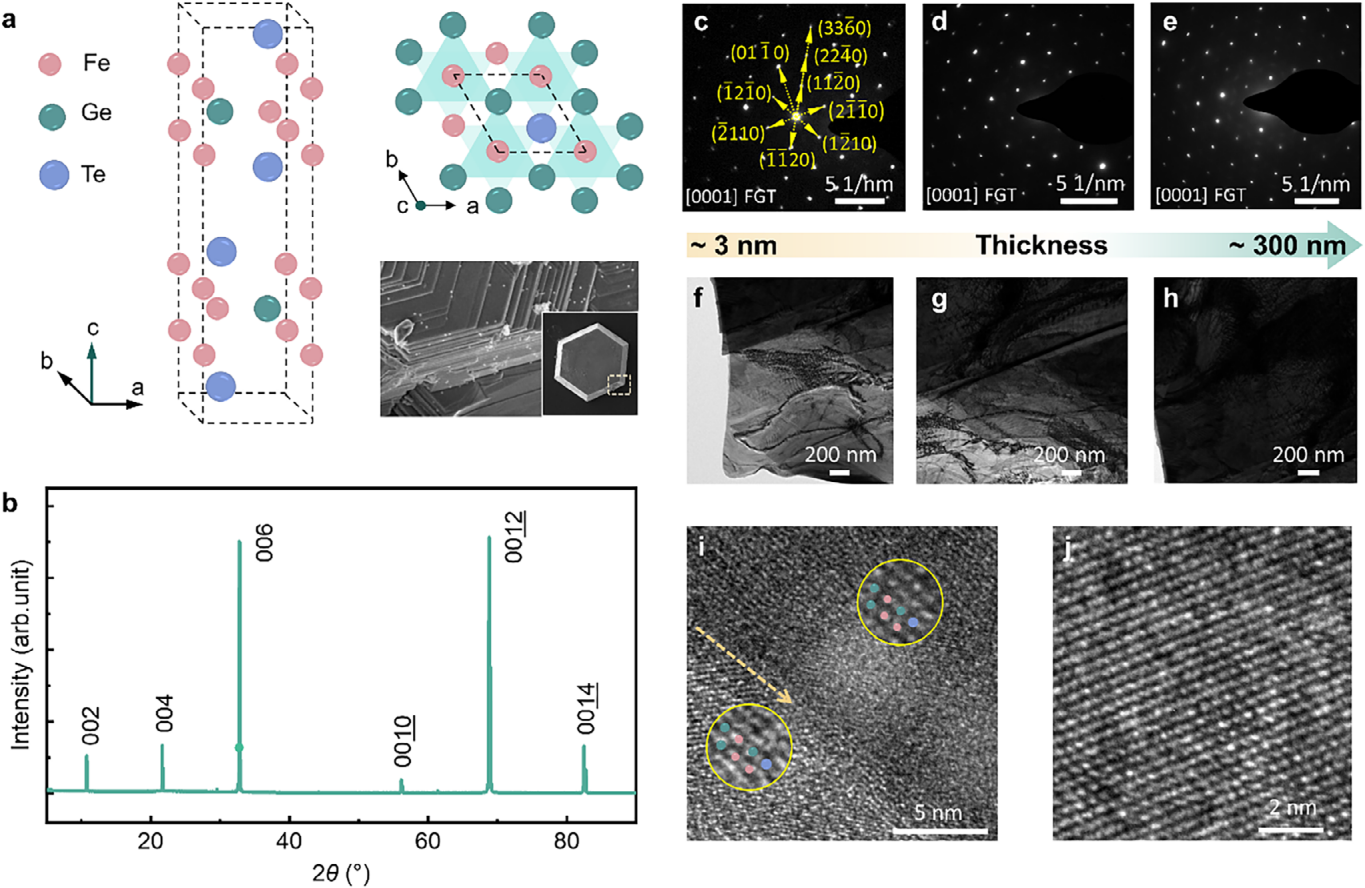
Structural properties of FGT crystals. a) Schematic diagram of the crystal structure of FGT. The lower right shows SEM images of bulk FGT. b) *X*‐ray diffraction spectrum of an FGT crystal. c–e) The [0001] zone axis electron diffraction patterns of FGT samples of varying thicknesses, from thin [3nm f)] to thick [300nm h)] ones. i) and j) High‐resolution TEM images taken from the ab planes of FGT flakes. Dashed lines indicate the positions of interlayer step edges, while solid lines in the zoomed‐in areas mark the continuity of lattice fringes across the interfaces, demonstrating the structural coherence and uniform stacking order.

The crystal structure of FGT is depicted in **Figure** **1**a. It crystallizes in the hexagonal system with space group P63/mmc and point group 6/m2/m2/m, known as *D*_6h_ in the Schönflies notation.^[^ ^33^ ^]^ Adjacent monolayers are stacked along the *c*‐axis via weak van der Waals forces, separated by an interlayer gap of ≈2.96 Å. Single‐crystal FGT is synthesized by the chemical vapor transport method. The element composition and bulk morphology are examined by energy‐dispersive spectroscopy and scanning electron microscopy, respectively. As shown in the lower right corner of Figure 1a, the scanning electron microscope (SEM) image reveals high crystallinity and a well‐cleaved layered morphology along the *ab* plane, consistent with the out‐of‐plane (002*n*) diffraction peaks observed in the X‐ray diffraction (XRD) pattern in Figure 1b, indicating excellent crystal orientation and phase purity. To further investigate the structural order across different thicknesses, transmission electron microscopy (TEM) is conducted on FGT flakes with varying thicknesses. Figure 1c–e display selected area electron diffraction (SAED) patterns acquired from corresponding regions in Figure 1f–h, consistently showing sharp and well‐defined diffraction spots. The thickness of these regions is estimated based on optical contrast observed during exfoliation, and the collected diffraction patterns represent an average over areas with varying thicknesses. The relatively clear diffraction spots observed in some thicker regions may be attributed to an increased scattering volume, possible dynamical diffraction effects, and variations in imaging conditions. These results confirm the single‐crystalline nature of the samples and indicate long‐range crystallographic coherence in the *ab*‐plane over lateral sizes ranging from hundreds of nanometers to micrometers. High‐resolution TEM images in Figure 1i,j reveal atomically ordered lattice fringes on both sides of interlayer step edges, highlighted by dashed lines, indicating that the lattice coherence is well‐preserved even across stacking interfaces. This observation is consistent with the SEM contrast shown in the inset of Figure 1a, where clear step edges and uniform terraces suggest orderly stacking and lateral crystallographic alignment. These results collectively confirm a high degree of structural coherence across multiple interfaces. Together, the HRTEM, SAED, and SEM data provide both local and statistical evidence for a well‐aligned layered structure with weak in‐plane anisotropy, in which each interlayer junction naturally forms an ASI. Such periodic ASIs constitute an intrinsic structural feature of FGT and lay the foundation for understanding the unusual magnetotransport behavior discussed below.

**Figure 2:**
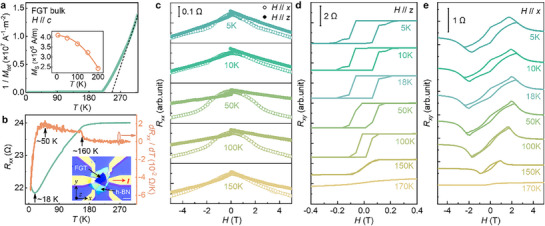
Magnetic and transport properties of FGT. a) Temperature dependence of 1/ *M_tot_* and *M*_S_ of bulk FGT, measured under a magnetic field applied along the *c*‐axis. b) *R_xx_* and *dR_xx_* / *dT* of the FGT device at different temperatures. The inset shows an optical microscope image of the fabricated device with a scale of 20 µm. c) MR measured with *H* // *x* (open circles) and *H* // *z* (solid circles). d,e) *R*_xy_ measured under magnetic fields applied along the *z*‐axis and *x*‐axis, respectively.

**Figure** **2**a shows the temperature dependence of the total magnetic moment (*M_tot_*) of bulk FGT, measured under an out‐of‐plane magnetic field (*H* = 0.1 T, parallel to the *c*‐axis). A rapid increase in 1/*M* suggests the magnetic phase transition of the bulk FGT crystal at ≈250 K. The saturation magnetization (*M*_S_), extracted from the magnetic hysteresis loops at different temperatures (shown in Figure S1, Supporting Information), is plotted in the inset. Through mechanical exfoliation and microfabrication,^[^ ^29^ ^]^ we prepare FGT devices with thicknesses ranging from 20 to 88 nm, as illustrated in the inset of Figure 2b. To prevent oxidation, all fabrication steps are conducted inside a glovebox, and the FGT nanoflakes are encapsulated with a hexagonal boron nitride (hBN) capping layer. The temperature‐dependent resistance of the FGT devices is shown in Figure 2b. Three characteristic temperatures are identified: a turning point in *R_xx_* at ≈18 K, a maximum in *dR_xx_* / *d*T at ≈50 K, and a kink point at ≈155 K. The MR of a representative FGT device is presented in Figure 2c, with *H* applied along both the *x*‐axis (current direction) and *z*‐axis (out‐of‐plane). The longitudinal resistance *R_xx_* is lower when *H* is parallel to the *c‐*axis, indicating a negative MR in FGT. The transverse resistance *R_xy_* as a function of magnetic field is shown in Figure 2d,e. When *H* is applied out of plane, the anomalous Hall loops in Figure 2d confirm the presence of perpendicular magnetic anisotropy.^[^ ^34^, ^35^ ^]^ When *H* // *x*, the *R_xy_* curve exhibits features characteristic of the topological Hall effect (THE),^[^ ^36^, ^37^ ^]^ a transport signal of chiral spin textures arising from mechanisms such as the Dzyaloshinskii‐Moriya interaction^[^ ^38^ ^]^ and the real‐space Berry phase.^[^ ^39^ ^]^ The THE signal saturates ≈5 T at 5 K, indicating that above this field strength, the magnetic moments in FGT are largely aligned with the external magnetic field. Therefore, in our angle‐dependent magnetoresistance measurements, a magnetic field of 8 T is employed to ensure full magnetization alignment and to minimize the influence of noncollinear spin textures.

**Figure 3:**
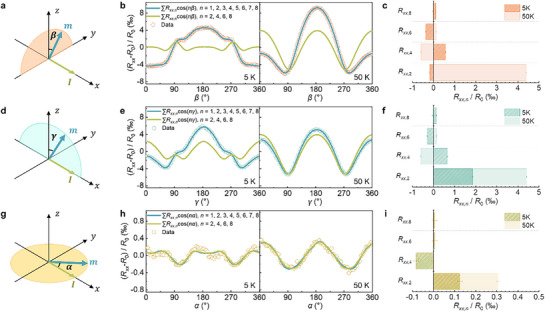
AMR of FGT devices across different rotation planes. a,d,g) depict the schematic configurations for angle‐dependent AMR measurements, corresponding to sample rotation within the *yz‐*, *xz‐*, and *xy*‐ planes, respectively. b,e,h) display the angular dependence of (*R_xx_* – *R*_0_)/*R*_0_, where *R*_0_ represents the intrinsic resistance of the device. The green curves represent fits including harmonic orders *n* = 1 to 8, while the yellow curves include only the even‐order components (*n* = 2, 4, 6, 8). Open circles represent the experimental data. c,f,i) The extracted normalized harmonic amplitudes *R_xx,n_*/*R*_0_ at 5 and 50 K.

Our research focuses on the variation of resistance in FGT devices as a function of the magnetic field angle (*β*, *γ*, and *α*) across the *yz‐*, *xz‐*, and *xy‐* planes, as shown in **Figure** **3**a–i, respectively. Due to slight deviations from ideal Hall bar geometry, the measured longitudinal resistance *R_xx_* may contain a minor contribution from the transverse resistance *R_xy_*, primarily reflected in the appearance of odd‐order angular harmonics. These components are attributed to subtle geometric asymmetries that are practically unavoidable in devices fabricated from mechanically exfoliated vdW flakes. Notably, the magnitude and sign of these odd‐order terms vary across different devices, indicating their non‐systematic nature and confirming that they do not originate from intrinsic transport mechanisms. Accordingly, our analysis focuses on the even‐order harmonic components of *R_xx_* (see S2, Supporting Information for details). *R_xx_* can be fitted using *R*_0_ + Σ*_n_R_xx_*_,_ *_n_* cos(*nθ*), where *θ* (= *α*, *β*, *γ*) is the magnetization angle when it rotates in the *xy*, *yz*, *zx* planes, respectively, *R*_0_ is the mean resistance of a device in a specific plane, and *R_xx_*_,_ *_n_* represents the *n*‐th symmetric component of *R_xx_* (note that we use the orthogonal set of functions, cos(*nθ*), rather than the non‐orthogonal set, cos*^n^θ*, for a more precise fit, as discussed in the Supporting Information). The AMR here is quantified as (*R_xx_*‐*R*_0_)/*R*_0_. By comparing the AMR measurements across the three planes, we identify three key features:

High‐order terms are observed, particularly for out‐of‐plane rotations (*yz‐* and *xz‐* planes) at 5 K. The 2nd, 4th, 6th, and 8th symmetric components of *R_xx_* are non‐negligible. These higher‐order UAMR observed in *yz‐*plane in a single ferromagnetic material deviate from the SMR theory,^[^ ^12^ ^]^ which applies to magnetic heterostructures and has only a cos*^2^β* symmetry. However, they are allowed by the two‐vector MR theory,^[^ ^25^, ^26^, ^27^ ^]^ which attributes the observed behavior to the coupling between magnetization and the ASI field. In this context, the ASI field plays a pivotal role in governing the angular‐dependent magnetoresistance, leading to the emergence of UAMR with higher‐order harmonics. This is in stark contrast to the conventional understanding based solely on spin‐current mechanisms, suggesting that ASI fields are the key determinant in systems like FGT, where atomic‐scale interfaces dominate the magnetotransport properties.

The out‐of‐plane UAMR is approximately one order of magnitude larger than the in‐plane UAMR. For example, Figure 3b shows ≈15‰ MR in the *yz*‐plane at 50K, while the traditional AMR in the *xy*‐plane is less than 1‰. Moreover, for out‐of‐plane configurations, the resistance at *γ* = 90^°^ is smaller than that at *γ* = 0^°^, indicating a larger resistance value when *H* lies in the *c*‐axis, which is perpendicular to the electrical current *I*. This means that, according to the traditional two‐current model, the out‐of‐plane UAMR observed here is “negative”.^[^ ^40^ ^]^ These results imply that the crystal structure of FGT is not the primary factor influencing AMR; instead, the ASI internal electric field plays a significant role in determining the out‐of‐plane AMR behavior. The dominance of the ASI field in these measurements suggests that it is not just the crystallographic symmetry of FGT, but the hidden interfacial fields that primarily govern the observed UAMR. This reinforces the notion that ASIs significantly influence magnetoresistance behaviors in vdW ferromagnets, going beyond conventional models focused on bulk material properties.

The higher‐order symmetric distributions of UAMR on the *yz‐*plane are similar to those on the *xz‐*plane, with the only notable difference being the sign of the 2nd‐order term *R_xx_*_,2_ at 5 K. Specifically, *R_xx_*_,2_/*R*_0_ = ‐0.17‰ (1.86‰) on the *yz‐* (*xz‐*) plane. As predicted by the two‐vector theory, $R_{xx}=r_{0}+r_{1}m_{x}^{2}+r_{2}m_{z}^{2}+r_{3}m_{z}^{4}+r_{4}m_{x}^{2}m_{z}^{2}$ and $R_{xy}=r_{5}m_{z}+r_{6}m_{z}^{3}+r_{1}m_{x}m_{y}+r_{4}m_{x}m_{y}m_{z}^{2}$, up to the 4th order in *m*, where $r_{i}\left(i=0∼6\right)$ are material parameters. Thus, the 2nd‐order term in the *yz*‐plane is $r_{2}m_{z}^{2}$, coming only from perpendicular field, while in the *xz*‐plane, it is $\left(r_{2}-r_{1}\right)m_{z}^{2}$, coming from both conventional AMR and perpendicular field. The sign changes imply *r*_1_ < *r*_2_ < 0. The relative importance of the two effects varies with the temperature, resulting in the sign change of *R_xx_*_,2_ at 5 K. These observations suggest that the contributions of the two effects are governed by the ASI field, which alters the balance between the perpendicular and in‐plane components of AMR as temperature changes.

**Figure 4:**
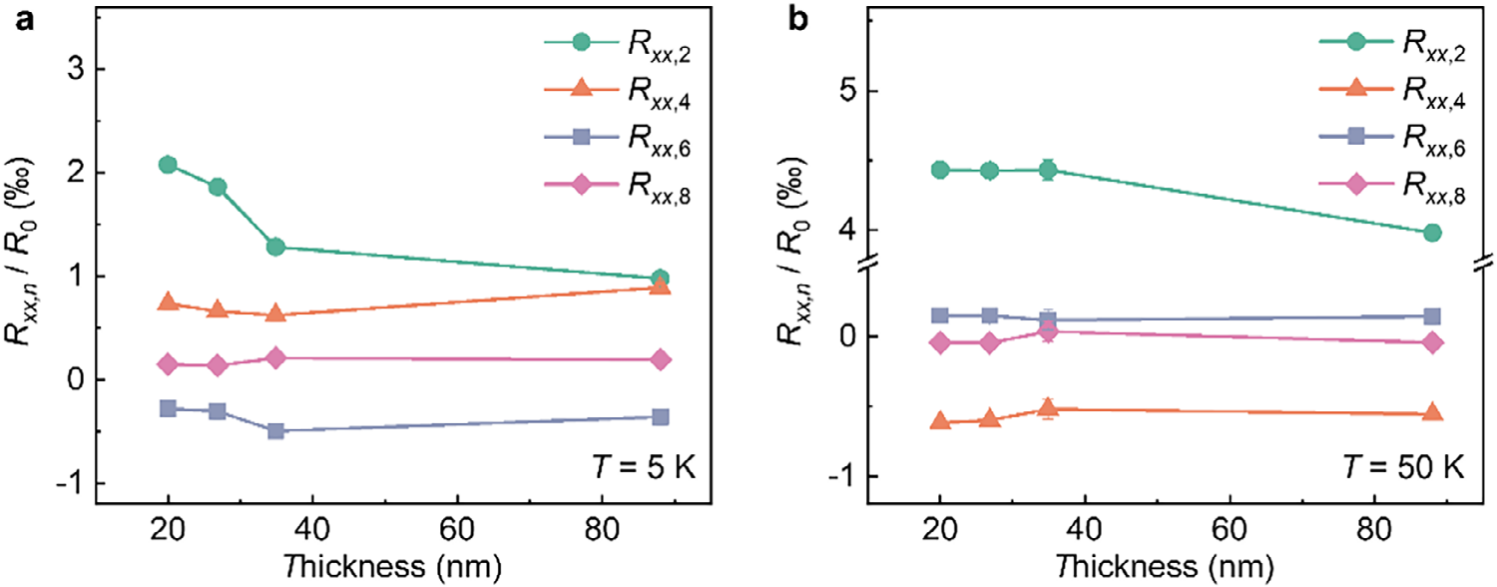
Thickness dependence of the extracted high‐order terms *R_xx_*_,_ *_n_* in the *xz*‐plane at a) *T* = 5 K and b) *T* = 50 K Solid lines serve as guides to the eye.

These features are universally observed across various devices. In sharp contrast to the high sensitivity of UAMR to film thickness in bilayers and single layers,^[^ ^27^ ^]^ the coefficients of high‐order terms do not exhibit significant thickness dependence, as shown in **Figure** **4**. This sharp difference agrees with two‐vector MR theory because UAMR in bilayers comes only from the electrons near the interface, while UAMR in vdW layered structures comes from all electrons in the sample. This is due to the nature of the inherently interfacial stacking, such that ASI fields can stably persist across FGT devices of varying thicknesses. Previous studies have suggested that deviations from twofold symmetry in AMR may arise from large demagnetizing fields and unsaturated magnetic moments.^[^ ^26^, ^27^, ^28^ ^]^ To address this possibility, we systematically compare the evolution of UAMR under different magnetic field strengths. Our results show that a magnetic field of 8 T is sufficient to fully saturate the magnetization of FGT, thereby excluding the influence of complex or unsaturated magnetic ground states on the observed AMR behavior (see Section S4, Supporting Information, for details).

**Figure 5:**
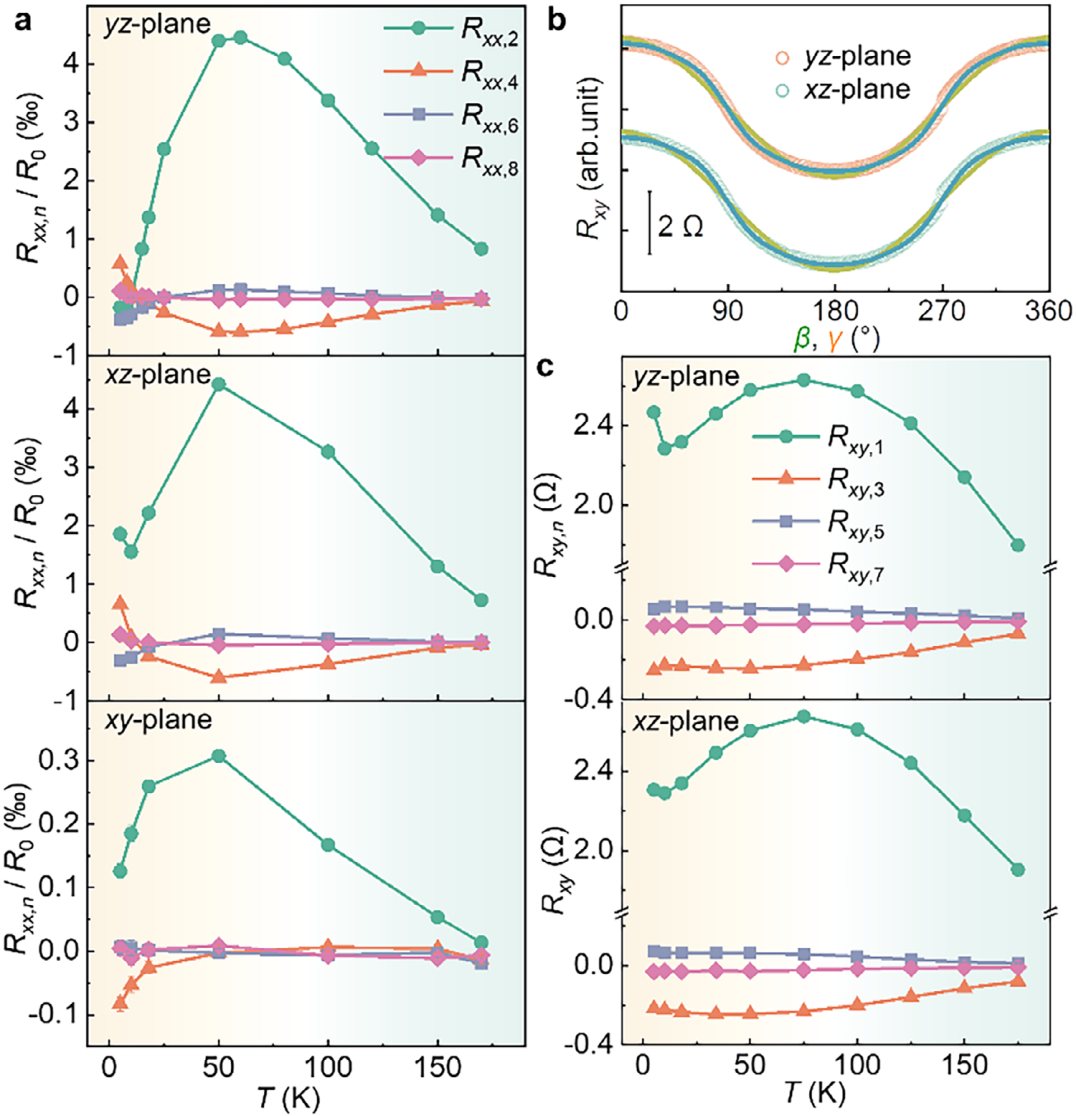
Temperature dependence of UAMR. a) Temperature dependence of the extracted high‐order terms in the UAMR. b) Variation of the transverse resistance *R_xy_* with magnetic field angles *β* (orange circles) and *γ* (green circles). Green lines are fitting curves that follow *R_xy_* ∝ Σ*_n_R_xy_*_,_ *_n_* cos(*nβ*) and Σ*_n_R_xy_*_,_ *_n_* cos(*nγ*), *n* = 1, 3, 5, 7, while yellow lines follow *R_xy_* ∝ Σ*_n_R_xy_*_,_ *_1_* cos(*β*) and Σ*_n_R_xy_*_,_ *_1_* cos(*γ*). The open circles denote the experimental data. c) Temperature dependence of the extracted orders in *R_xy_*.

Temperature dependencies of the extracted high‐order terms in the UAMR are depicted in **Figure** **5**a. The 6th and 8th order components of out‐of‐plane UAMR remain relatively large below 18 K but diminish rapidly at higher temperatures. The 4th‐order term exhibits a sign reversal ≈18 K, switching from positive to negative, reaching its maximal (negative) value near 50 K, and gradually decreasing toward zero at ≈155 K. In contrast, the 4th‐order in‐plane UAMR remains negative throughout and monotonically weakens with increasing temperature, vanishing above 50 K. The 2nd‐order AMR components in all three rotation planes peak near 50 K and subsequently decline with further temperature increase. The critical temperature points observed here—18, 50, and 155 K—coincide precisely with the anomalies in the temperature‐dependent longitudinal resistance curve shown in Figure 2b. These consistencies imply that the underlying transport mechanisms, including carrier types and scattering processes, may change significantly across different temperature regimes. We suggest that these variations are governed not solely by the crystal symmetry or magnetic anisotropy of FGT, but more fundamentally by the influence of ASI. The persistence and modulation of ASI‐induced internal electric fields throughout the layered vdW structure enable a strong and tunable coupling between itinerant electrons and localized moments. Previous reports have attributed the low‐temperature (< 18 K) anomalies to either an orbital two‐channel Kondo effect^[^ ^41^ ^]^ or to quantum corrections due to electron–electron coulomb interaction in the presence of Fe vacancies.^[^ ^42^ ^]^ A first‐order magnetic phase transition ≈155 K has also been reported in FGT flakes, attributed to competition between the perpendicular magnetic anisotropy and thermal fluctuations.^[^ ^35^ ^]^ However, our magnetization measurements do not reveal distinct anomalies at these temperatures, suggesting that electronic rather than magnetic origins, such as temperature‐driven changes in Fermi surface topology or scattering phase coherence, are more likely responsible. This reinforces the view that ASI dominate the emergence of UAMR, particularly in the out‐of‐plane configuration. Further studies, such as neutron scattering, are essential to elucidate the microscopic nature of these transitions.

To gain a comprehensive understanding of the electrical transport mechanisms, we further examine the angular dependence of the transverse resistance *R_xy_*, as illustrated in Figure 5b. In addition to the dominant first‐order components cos*β* and cos*γ*, we identify the presence of odd higher‐order harmonics in *R_xy_*, which can be quantitatively described by Σ*_n_R_xy_*_,_ *_n_* cos(*nθ*), where *R_xy_*_,_ *_n_* represents the *n*‐th symmetric component of *R_xy_*. The temperature dependence of the coefficients is presented in Figure 5c. In both the *yz‐* and *xz‐* planes, the distributions of the extracted *R_xy_* coefficients exhibit similar trends: *R_xy_*_,1_ peak near 75 K, while the third‐order term *R_xy_*_,3_ reaches a pronounced negative maximum ≈50 K. The fifth‐ and seventh‐order components remain relatively small and tend to vanish as the temperature approaches 155 K. The observation of these high‐order odd components in *R_xy_*, analogous to those in *R_xx_*, is incompatible with predictions from the conventional SMR theory, which typically yields only first‐order angular dependence. In contrast, these results are naturally captured within the framework of the two‐vector MR theory, which allows for such harmonic richness due to the interplay between the ASI field and the magnetization.

According to the two‐vector MR theory, intrinsic correlations exist between the angular dependencies of *R_xx_* and *R_xy_* across different rotation planes, which manifest as sum rules. These sum rules provide rigorous quantitative criteria for assessing the consistency between theoretical predictions and experimental observations. In our system, owing to the negligible magnetic anisotropy on the *xy‐*plane, the sum rules and other identities are:

(1) $${\left(R_{0}+\sum_{n}R_{\mathrm{xx},n}\right)}_{\mathrm{yz}}={\left(R_{0}+\sum_{n}R_{\mathrm{xx},n}\right)}_{\mathrm{xz}},n=2,4,6,8$$

(2) $${\left(R_{\mathrm{xy},n}\right)}_{\mathrm{yz}}={\left(R_{\mathrm{xy},n}\right)}_{\mathrm{xz}},n=1,3,5,7$$

Given that *R*_0_ with comparable values on the *yz*‐plane and *xz*‐plane is significantly greater than *R_xx_*_,2_, *R_xx_*_,4_, *R_xx_*_,6_, and *R_xx_*_,8_, the sum rule for the longitudinal magnetoresistance can be approximated as

(3) $$\sum_{n}{\left(\frac{R_{\mathrm{xx},n}}{R_{0}}\right)}_{\mathrm{yz}}=\sum_{n}{\left(\frac{R_{\mathrm{xx},n}}{R_{0}}\right)}_{\mathrm{xz}},n=2,4,6,8$$

**Figure 6:**
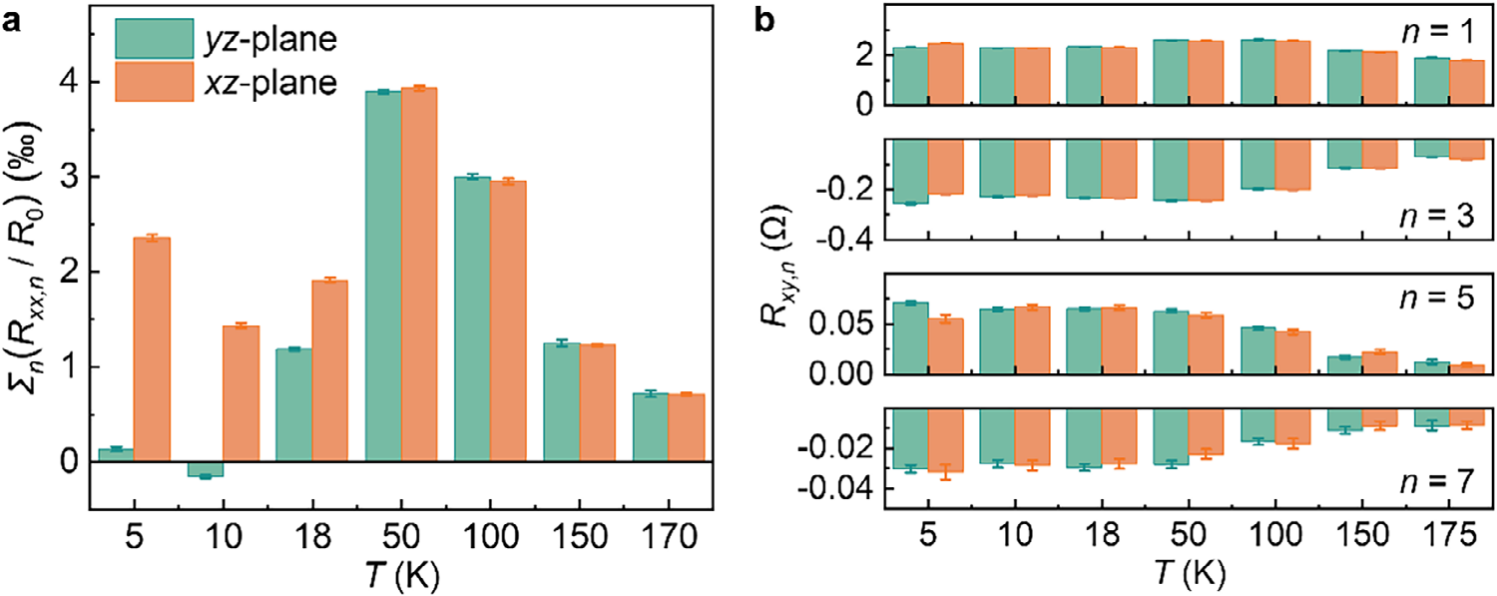
Verification of the sum rules predicted by the two‐vector MR theory. Temperature dependencies of a) Σ*_n_* (*R_xx_*_,_ *_n_* /*R_0_*) (*n* = 2, 4, 6, 8) and b) *R_xy,n_* (*n* = 1, 3, 5, 7) obtained at the *yz*‐plane (green) and *xz*‐plane (orange).

We compile Σ_n_(*R_xx,n_*/*R*_0_) (*n* = 2, 4, 6, 8) and *R_xy,n_* (*n* = 1, 3, 5, 7) at different temperatures, as depicted in **Figure** **6**a,b, respectively. Remarkably, Σ_n_(*R_xx,n_*/*R*_0_) obtained in the *yz‐*plane closely matches those in the *xz‐*plane when *T* ≥ 50 K, although noticeable deviations appear at lower temperatures. A similar trend is observed in *R_xy,n_*, where the coefficients from the *yz‐* and *xz‐*planes remain nearly identical for *T* ≥ 10K, with slight discrepancies at 5 K. These deviations at low temperatures likely originate from additional contributions to UAMR beyond the interfacial internal electric field, such as the crystallographic field, which becomes increasingly prominent at low temperatures due to the enhanced sensitivity of charge carriers to the local symmetry potential of the lattice.^[^ ^43^ ^]^

The good agreement between experiments and the two‐vector MR theory highlights the utility of this framework in describing angular‐dependent MR in systems with atomically sharp interfaces. This theory is grounded in general thermodynamic and symmetry principles, and posits that macroscopic observables, such as electrical resistivity, must be determined by the state variables that uniquely define a macroscopic state. In the context of layered magnetic systems, these vector state variables are magnetization and built‐in interfacial electric fields. These vectors constrain the symmetry‐allowed forms of the resistivity tensor, giving rise to specific angular harmonic components in MR, including the sum‐rule relationships that we experimentally verify. From this perspective, the two‐vector theory serves as a general and material‐agnostic phenomenological framework, independent of microscopic details such as spin‐orbit coupling or specific scattering mechanisms. Nevertheless, although the theory predicts the universal forms of UAMR, it does not provide quantitative predictions for the magnitude of each harmonic term. These amplitudes depend on system‐specific microscopic factors, including the band structure, disorder, temperature, etc. Furthermore, the current theory cannot apply to the angular dependence of MR in magnetic single crystals where there are at least four state variables, as briefly discussed in Section S6 (Supporting Information).

## 3 Conclusion

In summary, our study reveals that ASI internal electrical fields play a dominant role in dictating the UAMR behavior observed in vdW ferromagnet Fe_3_GeTe_2_. By leveraging a spin‐current‐free platform with ASI, we successfully disentangle the contributions of interfacial fields from conventional mechanisms and identify clear experimental signatures of ASI‐governed magnetotransport. These include: 1) a strongly enhanced out‐of‐plane UAMR—nearly an order of magnitude larger than its in‐plane counterpart; 2) the emergence of higher‐order angular harmonics up to the 8th order in resistance modulation; and 3) consistent verification of the harmonic sum rules predicted by the two‐vector magnetoresistance theory. Crucially, unlike UAMR in bilayer systems, which is highly sensitive to interfacial proximity and film thickness, the high‐order harmonic coefficients in Fe_3_GeTe_2_ exhibit negligible dependence on thickness. This provides compelling evidence that in layered vdW systems, the ASI fields are not confined to the immediate interface but persist across the entire stack, owing to the intrinsically interfacial nature of the vdW bonding architecture. These results establish ASI internal electric fields—not crystalline anisotropy or spin‐current effects—as the principal origin of the observed UAMR, and demonstrate that two‐vector MR theory offers a robust and quantitative framework for describing magnetoelectric coupling in vdW magnets. Broadly speaking, intrinsic ASI fields are a generic characteristic of many vdW magnetic systems. Thus, the same UAMR phenomena should exist across a broader class of materials beyond Fe_3_GeTe_2_. Our current understanding of UAMR in FGT provides a foundation for future investigations of systematic control and manipulation of UAMR by tuning interface configurations, interlayer coupling strength, and external control parameters such as gate voltage. Such efforts could enable a deeper understanding of interface‐driven phenomena in low‐dimensional magnetic systems and facilitate the development of vdW‐based spintronic platforms with tailored functionalities.

## 4 Experimental Section

### Sample Fabrication

Bulk Fe_3_GeTe_2_ (FGT) single crystals were synthesized via chemical vapor transport (CVT) using elemental precursors (Fe:Ge:Te = 3:1:2) with iodine transport agent. The sealed quartz ampoule was subjected to a temperature gradient from 750 to 680 °C in a two‐zone furnace for 7 days. The pristine FGT nanoflakes were mechanically exfoliated from bulk crystals using polydimethylsiloxane (PDMS) stamps within a nitrogen‐filled glove box. Selected nanoflakes were dry‐transferred onto SiO_2_/Si substrates pre‐patterned with bottom electrodes through viscoelastic stamping. The heterostructure was subsequently encapsulated with hexagonal boron nitride (hBN) via van der Waals assembly under optical microscopy guidance to prevent oxidation.

### Structural Characterization

The layered crystal structure of bulk FGT was analyzed by X‐ray diffraction (XRD) with Cu‐Kα radiation (λ = 1.5406 Å). To probe the in‐plane atomic arrangement of exfoliated FGT nanoflakes, mechanically cleaved flakes with thicknesses spanning 3–300 nm (determined by optical contrast) were transferred onto lacey carbon‐coated TEM grids using a dry transfer method. Aberration‐corrected high‐resolution transmission electron microscopy was performed at 200 kV accelerating voltage to resolve the ab‐plane lattice structure. Selected‐area electron diffraction patterns were concurrently acquired to confirm crystallographic orientation.

### Measurement of Magnetic Properties

The magnetic properties of bulk Fe_3_GeTe_2_ were investigated using a superconducting quantum interference device magnetometer. Temperature‐dependent magnetization was measured under an applied magnetic field *H* = 0.1 T aligned parallel to the crystallographic *c*‐axis. Magnetic hysteresis loops were acquired in field sweep mode at selected temperatures between 5 and 300 K during warming cycles. Prior to measurements, the sample orientation was confirmed via Laue diffraction to ensure precise alignment of the *c*‐axis with the magnetic field direction. Diamagnetic contributions from the sample holder were systematically subtracted using background measurements.

### Electrical Transport Measurements

The electrical transport properties were investigated using a Physical Property Measurement System. A current of 10*µA* was applied along the *x*‐direction within the ab‐plane of FGT, with the crystallographic *c*‐axis aligned perpendicular to the *z*‐direction. Angular‐dependent magnetoresistance measurements were performed by rotating an 8 T magnetic field within three orthogonal planes (*xy*‐, *yz*‐, and *xz*‐planes) while simultaneously recording the longitudinal resistance (*R_xx_*) and transverse resistance (*R_xy_*) as functions of the field orientation.

## Conflict of Interest

The authors declare no conflict of interest.

## Author Contributions

Q.C., J.W.S., and J.L. contributed equally to this work. Z.C.H., Z.M.Z., Y.Z., K.X., and X.R.W. supervised and led the project. J.L. and W.J. synthesized the Fe_3_GeTe_2_ crystals. Q.C and J.W. fabricated the device. J. L. and W.J. carried out magnetoelectricity measurements. J.W.S. performed the theoretical analysis. Z.P.Y. conducted TEM characterizations. All authors analyzed the data and contributed to manuscript preparation.

## Supporting information



Supporting Information

## Data Availability

The data that support the findings of this study are available from the corresponding author upon reasonable request.
