# Supplementary material for: Unusual Van der Waals Magnetoresistance in Stacked Ferromagnetic Fe3GeTe2: The Role of Atomically Sharp Interfaces
Source: Adv Sci (Weinh). 2025 Sep 12;12(42):e08244. doi: 10.1002/advs.202508244 (PMC12622409; doi:10.1002/advs.202508244)
Supplement: Supplementary file 1 — Supporting Information [file ADVS-12-e08244-s001.docx]

**Supplementary Materials for**

**Unusual Van der Waals Magnetoresistance in Stacked Ferromagnetic Fe_3_GeTe_2_: The Role of Atomically Sharp Interfaces**

Qian Chen^1,#^, Junwen Sun^2,#^, Jian Liang^1,#^, Wei Jiang^1^, Zhipeng Yu^4^, Zhaocong Huang^1^, Zhongming Zeng^4^, Ya Zhai^1,*^, Ke Xia^1,*^, Xiangrong Wang^2,3,*^

^1^ Key Laboratory of Quantum Materials and Devices of Ministry of Education, School of Physics, Southeast University, Nanjing, 211189, China

^2^ School of Science and Engineering, Chinese University of Hong Kong, Shenzhen, Shenzhen 51817, China

^3^ Department of Physics, The Hong Kong University of Science and Technology, Clear Water Bay, Kowloon, Hong Kong, China

^4^ Nanofabrication Facility, Suzhou Institute of Nano-Tech and Nano-Bionics, Chinese Academy of Sciences, Suzhou, Jiangsu 215123, China

**Contents**

**S1. Magnetic Hysteresis Loops of the** **Fe_3_GeTe_2_ Crystal**

**S2. Fitting Details of the High-order UAMR**

**S3. Reproducibility of the High-Order Anisotropic Magnetoresistance in Fe_3_GeTe_2_ Devices**

**S4. Magnetic Field Intensity Dependence of the Magnetoresistance**

**S5. Deriving UAMR Using the Two-vector Model**

**S6. Deriving UAMR Using the Four-vector Model**

**S1. Magnetic Hysteresis Loops of the Fe_3_GeTe_2_ Crystal**

**
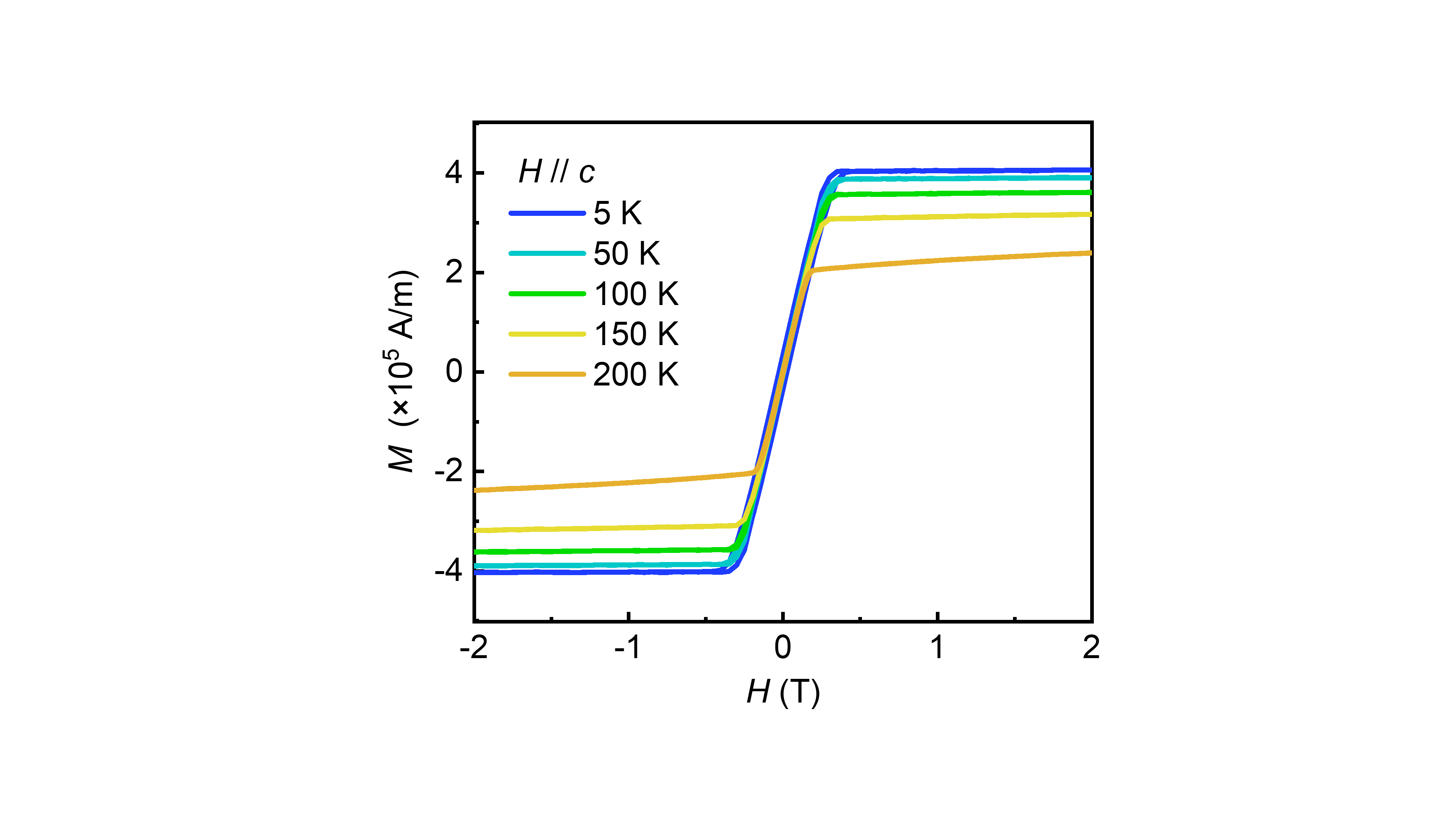
**

**FIG. S1** Hysteresis loops of the Fe_3_GeTe_2_ crystal with magnetic field applied along the *c*-axis.

Figure S1 shows the magnetic hysteresis loops of an Fe₃GeTe₂ (FGT) crystal with the magnetic field applied along the *c*-axis, measured at temperatures ranging from 5 K to 200 K. As the temperature increases, the saturation magnetization of the FGT crystal gradually decreases, as also illustrated in the inset of Fig. 2(a) in the main manuscript.

**S2. Fitting Details of the High-order UAMR**

**
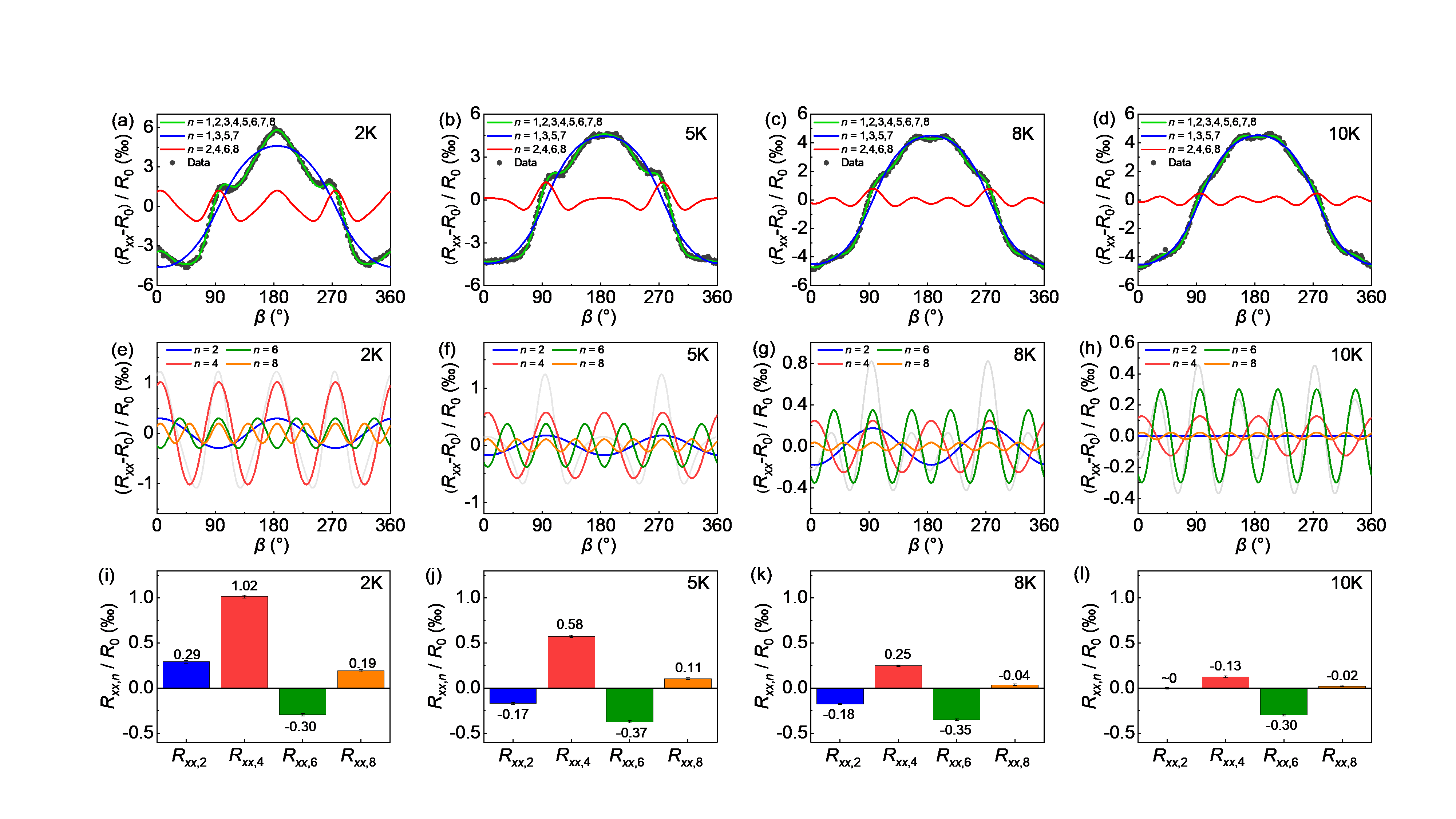
**

**FIG. S2** Fitting details of the high-order UAMR in *yz-*plane. The fitting function is given by *R_xx_* = *R*_0_ + Σ*_n_R_xx_*_,_*_n_*cos(*nβ*), and *R_xx_*_,_*_n_* represents the *n*-th symmetric component of *R_xx_*. In panels (a)–(d), the green curves represent fits including *n* = 1, 2, 3, 4, 5, 6, 7, 8，the blue curves include only the odd-order terms *n* = 1, 3, 5, 7, and the red curves include only the even-order terms *n* = 2, 4, 6, 8. Black dots correspond to the experimentally measured data points. In panels (e)–(h), the blue, red, green, and orange curves show the individual contributions of (*R_xx_*-*R*_0_)/*R*_0_ for *n* = 2, 4, 6, 8 extracted from the total even fits in panels (a)–(d). The fitting parameters *R_xx,n_* components normalized to *R*_0_ are shown in panels (i)–(l).

The UAMR quantified as (*R_xx_*-*R*_0_)/*R*_0_ here is fitted by *R_xx_* = *R*_0_ + Σ*_n_R_xx_*_,_*_n_*cos(*nθ*), where *θ* represents the angle of the magnetization in the *xy* (*α*), *yz* (*β*), *zx* (*γ*) planes relatively, and *R_xx_*_,_*_n_* represents the *n*-th symmetric component of *R_xx_*. Using the temperature points in the *yz*-plane as an example, the fitted curves are shown in Fig. S2(a)-(d). It can be observed that both odd (*n* = 1, 3, 5, 7) and even (*n* = 2, 4, 6, 8) symmetry components are present. This is because the device does not have a perfect Hall bar structure, leading to the measured longitudinal resistance *R_xx_* inevitably containing contributions from the transverse resistance *R_xy_*. These contributions can be separated using odd and even components, and we primarily focus on the contributions of the even terms to *R_xx_*. We have listed the contributions from the even terms in Fig. S2(e)-(h) and presented the coefficients of each component in Fig. S2(i)-(l). The same fitting methodology is applied to the other planes, as shown in Fig. S3.

**
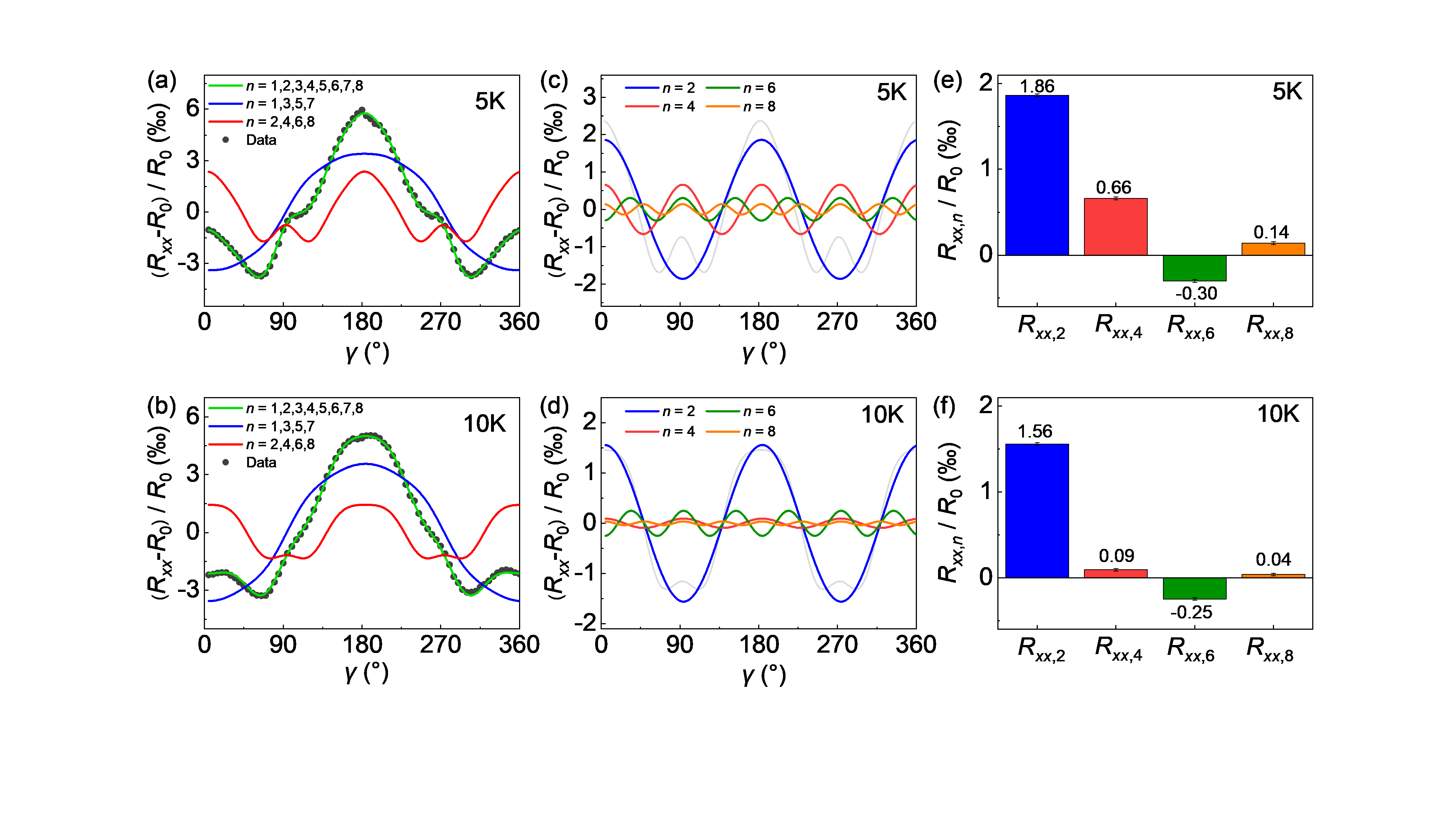
**

**FIG. S3** Fitting details of the high-order UAMR in *xz-*plane. The fitting function is given by *R_xx_* = *R*_0_ + Σ*_n_R_xx_*_,_*_n_*cos(*nγ*). In panels (a) and (b), the green curves represent fits including *n* = 1, 2, 3, 4, 5, 6, 7, 8，the blue curves include only the odd-order terms *n* = 1, 3, 5, 7, and the red curves include only the even-order terms *n* = 2, 4, 6, 8. Black dots correspond to the experimentally measured data points. In panels (c) and (d), the blue, red, green, and orange curves show the individual contributions of (*R_xx_*-*R*_0_)/*R*_0_ for *n* = 2, 4, 6, 8 extracted from the total even fits in panels (a) and (b). The fitting parameters *R_xx,n_* components normalized to *R*_0_ are shown in panels (e) and (f).

Here, we use $\cos nx$ instead of $\cos^{n} x$ for fitting, because $\cos nx$ forms an orthogonal set. As the number of fitting terms increases, the old coefficients remain almost unchanged. Therefore, even if a formula with fewer terms is used for fitting, the obtained coefficients are still relatively accurate. However, $\cos^{n} x$ fitting does not have these advantages because $\cos^{n} x$ is not an orthogonal set. As a result, when the number of fitting terms increases, the old coefficients will change significantly.

**S3. Reproducibility of the High-Order Anisotropic Magnetoresistance in FGT Devices**

**
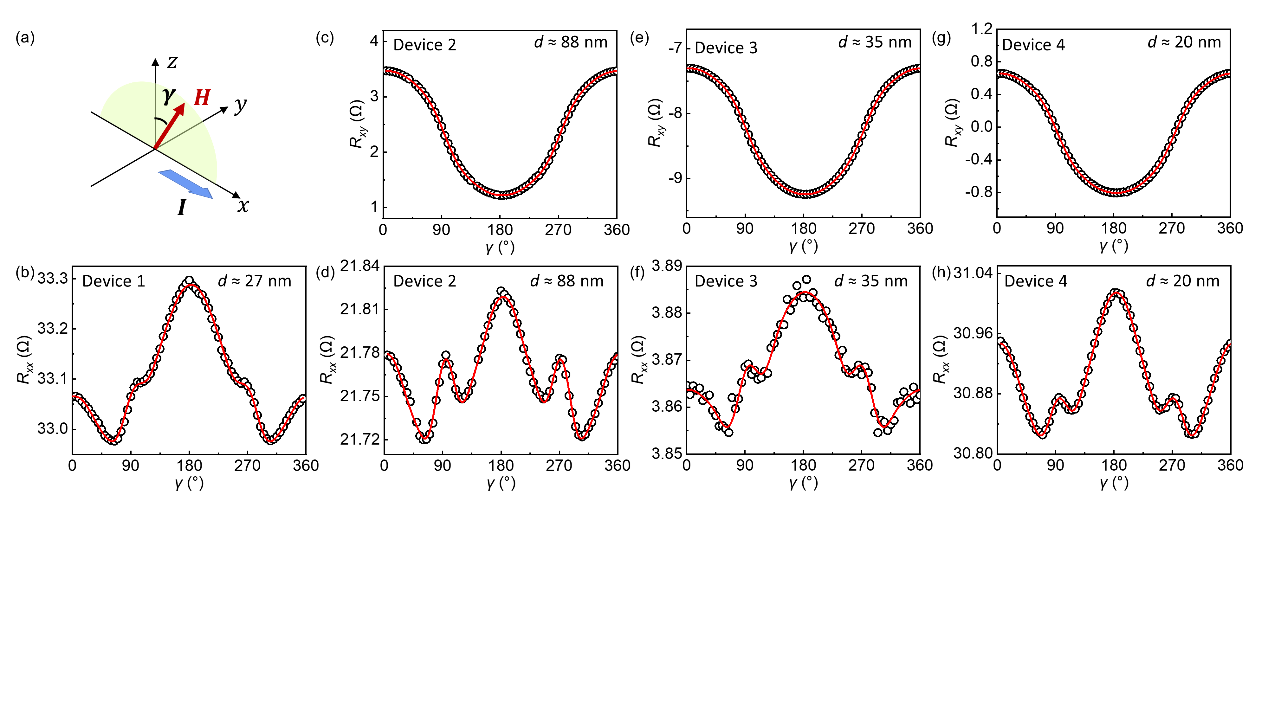
**

**FIG. S4** AMR measurements across FGT devices with different thickness *d*. (a) Schematic diagram of the measurement setup. (b) *R_xx_* for FGT device with *d* = 27 nm. (c) *R_xy_* and (d) *R_xx_* for FGT device with *d* = 88 nm. (e) *R_xy_* and (f) *R_xx_* for FGT device with *d* = 35 nm. (g) *R_xy_* and (h) *R_xx_* for FGT device with *d* = 20 nm. The open circles denote the experimental data. Red lines are fitting curves using the function *R_xx_* = *R*_0_ + Σ*_n_R_xx_*_,_*_n_*cos(*nγ*), *n* = 1, 2, 3, 4, 5, 6, 7, 8.

High-order UAMR is universally observed in various FGT devices, as shown in Fig. S4. We analyze the symmetry of UAMR in different devices at various temperatures, and extract the UAMR coefficients as a function of device thickness at 5 K and 50 K, as presented in Fig. 4. We find that the high-order terms do not exhibit significant thickness dependence, primarily due to the layered crystal structure of vdW magnets. This structure results in a proportional relationship between the number of interfaces and the thickness, thereby preventing the interface field from decreasing markedly with increasing thickness, as is typically observed in traditional single-interface magnetic heterostructures.

**S4. Magnetic Field Intensity Dependence of the Magnetoresistance**

To clarify the effects of the demagnetizing field and unsaturated magnetic moments on the UAMR, we compared the variations in UAMR under 5 T and 8 T, as shown in Fig. S5. It is found that an 8 T magnetic field is sufficient to saturate the magnetic moment of FGT, thereby ruling out complex magnetic ground states as an influence on AMR.

**
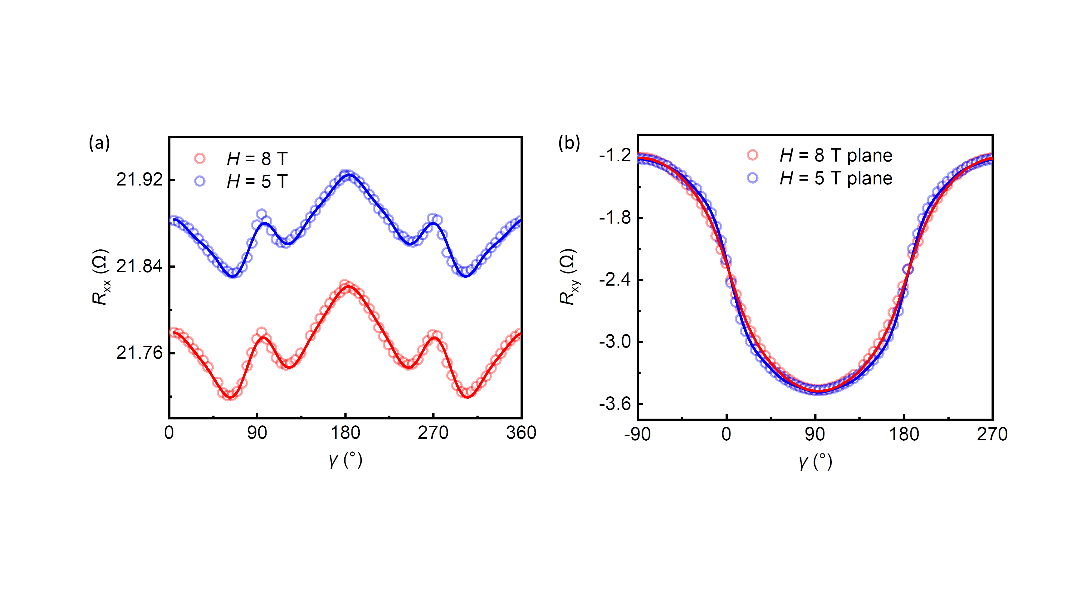
**

**FIG. S5** (a) Longitudinal resistance *R_xx_* and (b) transverse resistance *R_xy_* measured under magnetic fields of different strengths. Blue hollow circles and curves represent the experimental data and corresponding fit at *H* = 5 T, while red hollow circles and curves correspond to those measured at *H* = 8 T. The fitting function used is *R_xx_* = *R*_0_ + Σ*_n_R_xx_*_,_*_n_*cos(*nγ*), *n* = 1, 2, 3, 4, 5, 6, 7, 8. The close similarity between the results at 5 T and 8 T indicates that a magnetic field of 8 T is sufficient to fully saturate the magnetic moments in FGT, thereby excluding complex magnetic ground states as a contributing factor to the observed AMR.

**S5. Deriving UAMR using the two-vector model**

In FGT, in addition to magnetization $\vec{m}$, we consider a vector $\vec{n}$ representing the interface field. In the linear response region, the electric field $\vec{E}$ in response to an applied current density $\vec{J}$ is given by

$$\begin{aligned} \vec{E}=\rho\left( \vec{m},\vec{n} \right)\vec{J},\#\left( S.1 \right) \end{aligned}$$

where $\rho\left( \vec{m},\vec{n} \right)$ is the resistivity tensor of rank 2 that depends on the microscopic properties of the texture and the parameters that define its thermodynamic state. Regardless of the complexity of the microscopic interactions within the FGT, the tensor $\rho$ can be constructed only by the two vectors $\vec{m}$ and $\vec{n}$. Thus, $\rho$ should be the linear combination of $\vec{m}\vec{m},\vec{m}\vec{n}$, and $\vec{n}\vec{n}$. Each of the three Cartesian tensors is not irreducible, and can be decomposed into irreducible forms of a scalar, a vector, and a traceless symmetric tensor. For example, for the dyad $\vec{m}\vec{n}$, its effect on any vector $\vec{a}$ can be decomposed as follows[1]

$$\begin{aligned} \left( \vec{m}\vec{n} \right)\vec{a}=\frac{\vec{m}\cdot\vec{n}}{3}\vec{a}-\frac{1}{2}\left( \vec{m}\times\vec{n} \right)\times\vec{a}+\left( \frac{\vec{m}\vec{n}+\vec{n}\vec{m}}{2}\vec{a}-\frac{\vec{m}\cdot\vec{n}}{3}\vec{a} \right).\#\left( S.2 \right) \end{aligned}$$

Thus, from $\vec{m}$ and $\vec{n}$, it is possible to construct three vectors and three traceless symmetric tensors of ranks 2: $\vec{m}$, $\vec{n}$, $\vec{m}\times\vec{n}$, $\vec{m}\vec{m}-1/3$, $\vec{m}\vec{n}+\vec{n}\vec{m}-2\vec{m}\cdot\vec{n}/3$ and $\vec{n}\vec{n}-1/3$. With these angular dependent terms together with a scalar term, the electric field $\vec{E}$ due to $\vec{J}$, after grouping similar terms, must take the following generic form

$$\begin{aligned} \vec{E}=\rho\left( \vec{m},\vec{n} \right)\vec{J}=\rho_{0}\vec{J}+\left( B_{0}\vec{m}+B_{1}\vec{n}+B_{2}\vec{m}\times\vec{n} \right)\times\vec{J} \\ +A_{0}\left( \vec{J}\cdot\vec{m} \right)\vec{m}+A_{1}\left[ \left( \vec{J}\cdot\vec{m} \right)\vec{n}+\left( \vec{J}\cdot\vec{n} \right)\vec{m} \right]+A_{2}\left( \vec{J}\cdot\vec{n} \right)\vec{n},\#\left( S.2 \right) \end{aligned}$$

where $\rho_{0}$, $B_{0}$, $B_{1}$, $B_{2}$, $A_{0}$, $A_{1}$, and $A_{2}$, are parameters that depend on $\vec{m}\cdot\vec{n}=m_{z}$, which is the only scalar associated with the direction of $\vec{m}$, along with other material parameters that are independent of the direction of $\vec{m}$. Thus, the longitudinal resistivity is

$$\begin{aligned} \rho_{xx}=\frac{\vec{E}\cdot\hat{x}}{J}=\rho_{0}+A_{0}m_{x}^{2}, \end{aligned}$$

$$\begin{aligned} \rho_{xy}=\frac{\vec{E}\cdot\hat{y}}{J}=B_{0}m_{z}+B_{1}+A_{0}m_{x}m_{y}.\#\left( S.4 \right) \end{aligned}$$

Note that in the derivation of the above equation, we used $\vec{J}=J\hat{x}$. For crystals with reciprocity, $\vec{E}$ should remain unchanged under the transformation $\vec{n}\to-\vec{n}$. As a result, $\rho_{0}$ and $A_{0}$ must be even functions of $m_{z}$

$$\rho_{0}=\rho_{00}+\rho_{02}m_{z}^{2}+\rho_{04}m_{z}^{4}+\rho_{06}m_{z}^{6}+\rho_{06}m_{z}^{8}+\ldots,$$

$$A_{0}=A_{00}+A_{02}m_{z}^{2}+A_{04}m_{z}^{4}+A_{06}m_{z}^{6}+\ldots,$$

$$B_{0}=B_{00}+B_{02}m_{z}^{2}+B_{04}m_{z}^{4}+B_{06}m_{z}^{6}+\ldots,$$

$$\begin{aligned} B_{1}=B_{11}m_{z}+B_{13}m_{z}^{3}+B_{15}m_{z}^{5}+B_{17}m_{z}^{7}+\ldots.\#\left( S.5 \right) \end{aligned}$$

In the calculations that follow, we will retain terms up to $m_{i}^{8}$,

$$\rho_{xx}=\left( \rho_{00}+\rho_{02}m_{z}^{2}+\rho_{04}m_{z}^{4}+\rho_{06}m_{z}^{6}+\rho_{06}m_{z}^{8} \right)+\left( A_{00}+A_{02}m_{z}^{2}+A_{04}m_{z}^{4}+A_{06}m_{z}^{6} \right)m_{x}^{2},$$

$$\begin{aligned} \rho_{xy}=\left( B_{00}+B_{02}m_{z}^{2}+B_{04}m_{z}^{4}+B_{06}m_{z}^{6} \right)m_{z}+\left( B_{11}m_{z}+B_{13}m_{z}^{3}+B_{15}m_{z}^{5}+B_{17}m_{z}^{7} \right) \\ +\left( A_{00}+A_{02}m_{z}^{2}+A_{04}m_{z}^{4}+A_{06}m_{z}^{6} \right)m_{x}m_{y}. \#\left( S.6 \right) \end{aligned}$$

Considering the definitions of $\alpha,\beta$ and $\gamma$, we can substitute

$$\begin{aligned} \left[ \begin{aligned} m_{x} \\ m_{y} \\ m_{z} \end{aligned} \right]=\left[ \begin{aligned} \cos\alpha\\ \sin\alpha\\ 0 \end{aligned} \right],\left[ \begin{aligned} 0 \\ \sin\beta\\ \cos\beta\end{aligned} \right],\left[ \begin{aligned} \sin\gamma\\ 0 \\ \cos\gamma\end{aligned} \right].\#\left( S.7 \right) \end{aligned}$$

This leads to the following expressions

$$\rho_{xx}\left( \alpha\right)=\rho_{1}+\rho_{2}\cos2\alpha,$$

$$\rho_{xx}\left( \beta\right)=\rho_{3}+\rho_{4}\cos2\beta+\rho_{5}\cos4\beta+\rho_{6}\cos6\beta+\left( \rho_{1}-\rho_{2}-\rho_{3}+\rho_{4}-\rho_{5}+\rho_{6} \right)\cos8\beta,$$

$$\rho_{xx}\left( \gamma\right)=\rho_{7}+\rho_{8}\cos2\gamma+\rho_{9}\cos4\gamma+\left( -\rho_{2}+\rho_{4}+\rho_{6}-\rho_{8} \right)\cos6\gamma+\left( \rho_{1}+\rho_{4}+\rho_{6}-\rho_{7}-\rho_{9} \right)\cos8\gamma,$$

$$\rho_{xy}\left( \alpha\right)=\rho_{2}\sin2\alpha,$$

$$\rho_{xy}\left( \beta\right)=\rho_{10}\cos\beta+\rho_{11}\cos3\beta+\rho_{12}\cos5\beta+\rho_{13}\cos7\beta,$$

$$\begin{aligned} \rho_{xy}\left( \gamma\right)=\rho_{10}\cos\gamma+\rho_{11}\cos3\gamma+\rho_{12}\cos5\gamma+\rho_{13}\cos7\gamma.\#\left( S.8 \right) \end{aligned}$$

The expressions for $\rho_{1}$ to $\rho_{13}$ are as follows

$$\rho_{1}=\frac{1}{2}\left( A_{00}+2\rho_{00} \right),\rho_{2}=\frac{1}{2}A_{00},\rho_{3}=\frac{1}{128}\left( 128\rho_{00}+64\rho_{02}+48\rho_{04}+40\rho_{06}+35\rho_{08} \right),$$

$$\rho_{4}=\frac{1}{128}\left( 64\rho_{02}+64\rho_{04}+60\rho_{06}+56\rho_{08} \right),$$

$$\rho_{5}=\frac{1}{128}\left( 16\rho_{04}+24\rho_{06}+28\rho_{08} \right),\rho_{6}=\frac{1}{128}\left( 4\rho_{06}+8\rho_{08} \right),$$

$$\rho_{7}=\frac{1}{128}\left( 64A_{00}+16A_{02}+8A_{04}+5A_{06}+128\rho_{00}+64\rho_{02}+48\rho_{04}+40\rho_{06}+35\rho_{08} \right),$$

$$\rho_{8}=\frac{1}{128}\left( -64A_{00}+4A_{04}+4A_{06}+64\rho_{02}+64\rho_{04}+60\rho_{06}+56\rho_{08} \right),$$

$$\rho_{9}=\frac{1}{128}\left( -16A_{02}-8A_{04}-4A_{06}+16\rho_{04}+24\rho_{06}+28\rho_{08} \right),$$

$$\rho_{10}=\frac{1}{64}\left( 64B_{00}+48B_{02}+40B_{04}+35B_{06}+64B_{11}+48B_{13}+40B_{15}+35B_{17} \right),$$

$$\rho_{11}=\frac{1}{64}\left( 16B_{02}+20B_{04}+21B_{06}+16B_{13}+20B_{15}+21B_{17} \right),$$

$$\begin{aligned} \rho_{12}=\frac{1}{64}\left( 4B_{04}+7B_{06}+4B_{15}+7B_{17} \right),\rho_{13}=\frac{1}{64}\left( B_{06}+B_{17} \right).\#\left( S.9 \right) \end{aligned}$$

From the expressions of $\rho_{xx}$ and $\rho_{xy}$, it can be seen that $\rho_{xx}\left( \alpha\right)$ and $\rho_{xy}\left( \alpha\right)$ only have second-order symmetry, $\rho_{xx}\left( \beta\right)$ and $\rho_{xx}\left( \gamma\right)$ only have even-order symmetry, and $\rho_{xy}\left( \beta\right)$ and $\rho_{xy}\left( \gamma\right)$ only have odd-order symmetry. In addition, we can also derive the following rules:

$$\sum_{k=0} \rho_{xx,2k}\left( \beta\right)=\sum_{k=0} \rho_{xx,2k}\left( \gamma\right)$$

$$\begin{aligned} \rho_{xy,2k+1}\left( \beta\right)=\rho_{xy,2k+1}\left( \gamma\right) for every k\geq0\#\left( S.10 \right) \end{aligned}$$

where $\rho_{xx,k}$ represents the coefficient of the $k$-fold symmetry in $\rho_{xx,k}$, and the same applies to $\rho_{xy,k}$. It should be noted that, considering the following characteristics of the actual data

$$\begin{aligned} \rho_{xx,0}\left( \beta\right)\gg\rho_{xx,2k}\left( \beta\right), \rho_{xx,0}\left( \gamma\right)\gg\rho_{xx,2k}\left( \gamma\right) k=1,2,\ldots, \\ \rho_{xx,0}\left( \beta\right)\approx\rho_{xx,0}\left( \gamma\right),\#\left( S.11 \right) \end{aligned}$$

we can approximate

$$\begin{aligned} \frac{\sum_{k=1} \rho_{xx,2k}\left( \beta\right)}{\rho_{xx,0}\left( \beta\right)}\approx\frac{\sum_{k=1} \rho_{xx,2k}\left( \gamma\right)}{\rho_{xx,0}\left( \gamma\right)}.\#\left( S.12 \right) \end{aligned}$$

The relative error of the above approximation is given by

$$\begin{aligned} \varepsilon=\frac{\left| \sum_{k=1}^{n} \frac{\rho_{xx,2k}\left( \beta\right)}{\rho_{xx,0}\left( \beta\right)}-\sum_{k=1}^{n} \frac{\rho_{xx,2k}\left( \gamma\right)}{\rho_{xx,0}\left( \gamma\right)} \right|}{\left| \sum_{k=1}^{n} \frac{\rho_{xx,2k}\left( \beta\right)}{\rho_{xx,0}\left( \beta\right)} \right|}=\frac{\left| O\left( \rho_{xx,0}\left( \beta\right)-\rho_{xx,0}\left( \gamma\right) \right) \right|}{\left| \sum_{k=1}^{n} \rho_{xx,2k}\left( \beta\right) \right|}.\#\left( S.13 \right) \end{aligned}$$

Substituting the actual fitting values, we find $\varepsilon\approx0.001/0.02=5\%$. Therefore, the deviation of the sum rule at low temperatures in the article is physical, rather than a non-physical result caused by data errors.

**S6. Deriving UAMR Using the Four-vector Model**

It is important to emphasize that we have completely ignored the effects of the in-plane crystal axes, $\vec{a}$ and $\vec{b}$, in our discussion. At low temperatures, since the conduction electrons are primarily confined to the Fermi surface, their response to the crystal axes becomes more sensitive compared to higher temperatures. Therefore, the influence of the crystal axes must be taken into account under these conditions. When these factors are taken into account, $\rho$ becomes a function of $\vec{m},\vec{n},\vec{a}$ and $\vec{b}$, i.e., $\rho=\rho\left( \vec{m},\vec{n},\vec{a},\vec{b} \right)$. Consequently, both $\rho_{\mathrm{xx}}$, $\rho_{\mathrm{xy}}$ as well as the sum rule, will undergo changes accordingly. At this point, equation (S.1) needs to be modified to[2]

$$\begin{aligned} \vec{E}=\rho_{0}\vec{J}+\left( B_{0}\vec{m}+B_{1}\vec{n}+B_{2}\vec{m}\times\vec{n}+\boldsymbol{B}_{\boldsymbol{3}}\vec{\boldsymbol{a}}\boldsymbol{+}\boldsymbol{B}_{\boldsymbol{4}}\vec{\boldsymbol{m}}\boldsymbol{\times}\vec{\boldsymbol{a}}\boldsymbol{+}\boldsymbol{B}_{\boldsymbol{5}}\vec{\boldsymbol{b}}\boldsymbol{+}\boldsymbol{B}_{\boldsymbol{6}}\vec{\boldsymbol{m}}\boldsymbol{\times}\vec{\boldsymbol{b}} \right)\times\vec{J} \\ +A_{0}\left( \vec{J}\cdot\vec{m} \right)\vec{m}+A_{1}\left[ \left( \vec{J}\cdot\vec{m} \right)\vec{n}+\left( \vec{J}\cdot\vec{n} \right)\vec{m} \right]+A_{2}\left( \vec{J}\cdot\vec{n} \right)\vec{n} \\ +\boldsymbol{A}_{\boldsymbol{3}}\left[ \left( \vec{\boldsymbol{J}}\boldsymbol{\cdot}\vec{\boldsymbol{m}} \right)\vec{\boldsymbol{a}}\boldsymbol{+}\left( \vec{\boldsymbol{J}}\boldsymbol{\cdot}\vec{\boldsymbol{a}} \right)\vec{\boldsymbol{m}} \right]\boldsymbol{+}\boldsymbol{A}_{\boldsymbol{4}}\left( \vec{\boldsymbol{J}}\boldsymbol{\cdot}\vec{\boldsymbol{a}} \right)\vec{\boldsymbol{a}}\boldsymbol{+}\boldsymbol{A}_{\boldsymbol{5}}\left[ \left( \vec{\boldsymbol{J}}\boldsymbol{\cdot}\vec{\boldsymbol{m}} \right)\vec{\boldsymbol{b}}\boldsymbol{+}\left( \vec{\boldsymbol{J}}\boldsymbol{\cdot}\vec{\boldsymbol{b}} \right)\vec{\boldsymbol{m}} \right]\boldsymbol{+}\boldsymbol{A}_{\boldsymbol{6}}\left( \vec{\boldsymbol{J}}\boldsymbol{\cdot}\vec{\boldsymbol{b}} \right)\vec{\boldsymbol{b}} \\ \boldsymbol{+}\boldsymbol{A}_{\boldsymbol{7}}\left[ \left( \vec{\boldsymbol{J}}\boldsymbol{\cdot}\vec{\boldsymbol{n}} \right)\vec{\boldsymbol{a}}\boldsymbol{+}\left( \vec{\boldsymbol{J}}\boldsymbol{\cdot}\vec{\boldsymbol{a}} \right)\vec{\boldsymbol{n}} \right]\boldsymbol{+}\boldsymbol{A}_{\boldsymbol{8}}\left[ \left( \vec{\boldsymbol{J}}\boldsymbol{\cdot}\vec{\boldsymbol{n}} \right)\vec{\boldsymbol{b}}\boldsymbol{+}\left( \vec{\boldsymbol{J}}\boldsymbol{\cdot}\vec{\boldsymbol{b}} \right)\vec{\boldsymbol{n}} \right]+\boldsymbol{A}_{\boldsymbol{9}}\left[ \left( \vec{\boldsymbol{J}}\boldsymbol{\cdot}\vec{\boldsymbol{a}} \right)\vec{\boldsymbol{b}}\boldsymbol{+}\left( \vec{\boldsymbol{J}}\boldsymbol{\cdot}\vec{\boldsymbol{b}} \right)\vec{\boldsymbol{a}} \right],\#\left（ S.14 \right） \end{aligned}$$

the boldfaced term arises from taking into account the two order parameters $\vec{a}$ and $\vec{b}$. Similar to (S.5), $\rho_{0}$, $A_{\alpha}$ and $B_{\beta}$ depend on the dot products of $\vec{m}$ and $\vec{n}$, $\vec{a}$ and $\vec{b}$, (ie., $\vec{m}\cdot\vec{n}=m_{n},\vec{m}\cdot\vec{a}=m_{a},\vec{m}\cdot\vec{b}=m_{b}$), and can be expressed as polynomials as follows

$$\rho_{0}=\sum_{i,j,k=0} \rho_{0ijk}m_{n}^{i}m_{a}^{j}m_{b}^{k}$$

$$A_{\alpha}=\sum_{i,j,k=0} A_{\alpha ijk}m_{n}^{i}m_{a}^{j}m_{b}^{k}$$

$$\begin{aligned} B_{\beta}=\sum_{i,j,k=0} B_{\beta ijk}m_{n}^{i}m_{a}^{j}m_{b}^{k}\#\left（ S.15 \right） \end{aligned}$$

We can immediately observe that, since $\rho_{0ijk},A_{\alpha ijk}$ and $B_{\beta ijk}$ are in fact the coefficients in the Taylor expansion, it follows that

$$\rho_{0ijk}=\rho_{0\sigma\left( i \right)\sigma\left( j \right)\sigma\left( k \right)}$$

$$A_{\alpha ijk}=A_{\alpha\sigma\left( i \right)\sigma\left( j \right)\sigma\left( k \right)}$$

$$\begin{aligned} B_{\beta ijk}=B_{\beta\sigma\left( i \right)\sigma\left( j \right)\sigma\left( k \right)}\#\left( S.16 \right) \end{aligned}$$

Here, $\sigma$ denotes the permutation of $i,j$ and $k$.

It is worth noting that, since we now consider the three order parameters $\vec{n}$, $\vec{a}$ and $\vec{b}$, the symmetry of the system becomes more complex than in the case where only $\vec{n}$ was considered. We can utilize the Neumann principle to fully take into account the influence of symmetry on the tensor. According to the Neumann principle, the components of the property tensor (which relates the excitation and the response) remain unchanged in any two coordinate systems that are related by a symmetry operation. For example, for a second-rank property tensor $T_{ij}$, the Neumann principle says

$$\begin{aligned} T_{ij}=T_{ij}^{'}\#\left( S.17 \right) \end{aligned}$$

Here, $T_{ij}$ are the tensor components in the $xyz$ coordinate system, while $T_{ij}^{'}$ are the components in the $x^{'}y^{'}z^{'}$ coordinate system. Note that

$$\begin{aligned} T_{ij}^{'}=R_{ip}R_{jq}T_{pq}\#\left( S.18 \right) \end{aligned}$$

where $R$ is the matrix representation of the symmetry operation relate $xyz$ and $x^{'}y^{'}z^{'}$, therefore we have

$$\begin{aligned} T_{ij}=R_{ip}R_{jq}T_{pq}\#\left( S.19 \right) \end{aligned}$$

The above equation demonstrates how the symmetry of the system constrains the property tensor. In practice, it is not necessary to apply $R$ over all elements of the symmetry group; it suffices to consider only the generators of the symmetry group. The generator matrices of the crystal point group of FGT are (the Seitz notation is used here): $3_{001}^{+}$, which represents a counterclockwise rotation of 60° around the [001] axis; $2_{001}$, which represents a mirror reflection with respect to the (001) crystal plane; $2_{110}$, which represents a mirror reflection with respect to the (110) crystal plane；and $-1$, which represents a spatial inversion operation. Since $\rho_{0ijk},A_{\alpha ijk}$ and $B_{\beta ijk}$ are all components of the property tensor, we can apply the Neumann principle to them.

Taking into account the constraints imposed on the tensor by Eqs. (S.16) and (S.19), and by further calculating $\rho_{\mathrm{xx}}$ and $\rho_{\mathrm{xy}}$, we obtain

$$\rho_{xx}^{\theta}\left( \alpha\right)=\rho_{1}+\rho_{2}\cos2\alpha+\rho_{3}\cos6\theta\cos4\alpha-\rho_{3}\sin6\theta\sin4\alpha+\rho_{4}\cos6\theta\cos6\alpha-\rho_{4}\sin6\theta\sin6\alpha,$$

$$\rho_{xx}^{\theta}\left( \beta\right)=\rho_{5}+\rho_{6}\cos6\theta+\rho_{7}\sin6\theta\cos\beta-\frac{3}{2}\rho_{7}\sin6\theta\cos3\beta+\frac{1}{2}\rho_{7}\sin6\theta\cos5\beta+\left( \left( -\frac{5\rho_{3}}{16}+\frac{5\rho_{4}}{16}-\frac{\rho_{6}}{2} \right)\cos6\theta+\rho_{8} \right)\cos2\beta+\left( \left( \frac{\rho_{3}}{2}-\frac{\rho_{4}}{2}-\rho_{6} \right)\cos6\theta+\rho_{9} \right)\cos4\beta+\left( -\rho_{1}+\rho_{2}+\rho_{5}-\rho_{8}+\rho_{9}+\left( -\frac{3\rho_{3}}{16}+\frac{3\rho_{4}}{16}+\frac{\rho_{6}}{2} \right)\cos6\theta\right)\cos6\beta,$$

$$\rho_{xx}^{\theta}\left( \gamma\right)=\rho_{10}+\left( \frac{5\rho_{4}}{8}+\rho_{6} \right)\cos6\theta+\rho_{7}\sin6\theta\cos\gamma-\frac{3}{2}\rho_{7}\sin6\theta\cos3\gamma+\frac{1}{2}\rho_{7}\sin6\theta\cos5\gamma+\left( \rho_{11}+\left( -\frac{5\rho_{3}}{16}-\frac{5\rho_{4}}{8}-\frac{\rho_{6}}{2} \right)\cos6\theta\right)\cos2\gamma+\left( \rho_{2}+\rho_{5}+\rho_{9}-\rho_{10}+\left( \frac{\rho_{3}}{2}-\frac{\rho_{4}}{8}-\rho_{6} \right)\cos6\theta\right)\cos4\gamma+\left( -\rho_{1}+\rho_{5}+\rho_{9}-\rho_{11}+\left( -\frac{3\rho_{3}}{16}+\frac{\rho_{4}}{8}+\frac{\rho_{6}}{2} \right)\cos6\theta\right)\cos6\gamma,$$

$$\rho_{xy}^{\theta}\left( \alpha\right)=\rho_{2}\sin2\alpha-\rho_{3}\cos6\theta\sin4\alpha-\rho_{3}\sin6\theta\cos4\alpha+\rho_{12}\cos6\theta\sin6\alpha+\rho_{12}\sin6\theta\cos6\alpha,$$

$$\rho_{xy}^{\theta}\left( \beta\right)=\left( -\frac{5\rho_{4}}{16}-\rho_{6}-\frac{5\rho_{12}}{16} \right)\sin6\theta+\left( \rho_{7}\cos6\theta+\rho_{13} \right)\cos\beta+\left( -\frac{3}{2}\rho_{7}\cos6\theta+\rho_{14} \right)\cos3\beta+\left( \frac{1}{2}\rho_{7}\cos6\theta+\rho_{15} \right)\cos5\beta+\left( -\frac{\rho_{3}}{2}+\frac{5\rho_{4}}{16}+\rho_{6}-\frac{3\rho_{12}}{16} \right)\sin6\theta\cos4\beta+\left( \frac{3\rho_{3}}{16}-\frac{5\rho_{4}}{32}-\frac{\rho_{6}}{2}+\frac{\rho_{12}}{32} \right)\sin6\theta\cos6\beta+\left( \frac{5\rho_{3}}{16}+\frac{5\rho_{4}}{32}+\frac{\rho_{6}}{2}+\frac{15\rho_{12}}{32} \right)\sin6\theta\cos2\beta,$$

$$\begin{aligned} \rho_{xy}^{\theta}\left( \gamma\right)=\left( -\frac{5\rho_{4}}{16}-\rho_{6}+\frac{5\rho_{12}}{16} \right)\sin6\theta+\left( \rho_{7}\cos6\theta+\rho_{16} \right)\cos\gamma+\left( -\frac{3}{2}\rho_{7}\cos6\theta+\rho_{17} \right)\cos3\gamma\\ +\left( \frac{1}{2}\rho_{7}\cos6\theta+\rho_{13}+\rho_{14}+\rho_{15}-\rho_{16}-\rho_{17} \right)\cos5\gamma\\ +\left( \frac{5\rho_{3}}{16}+\frac{5\rho_{4}}{32}+\frac{\rho_{6}}{2}-\frac{15\rho_{12}}{32} \right)\sin6\theta\cos2\gamma\\ +\left( -\frac{\rho_{3}}{2}+\frac{5\rho_{4}}{16}+\rho_{6}+\frac{3\rho_{12}}{16} \right)\sin6\theta\cos4\gamma\\ +\left( \frac{3\rho_{3}}{16}-\frac{5\rho_{4}}{32}-\frac{\rho_{6}}{2}-\frac{\rho_{12}}{32} \right)\sin6\theta\cos6\gamma. \#\left( S.20 \right) \end{aligned}$$

Unlike the two-vector model, in this case, $\rho_{xx}\left( \alpha\right)$ and $\rho_{xy}\left( \alpha\right)$ exhibit higher-order even symmetry, while $\rho_{xx}\left( \beta,\gamma\right)$ and $\rho_{xy}\left( \beta,\gamma\right)$ possess both even-order and odd-order symmetry. Moreover, the rule we previously obtained in the two-vector model is now updated as follows:

$$\sum_{k=0} \rho_{xx,2k}\left( \beta\right)-\sum_{k=0} \rho_{xx,2k}\left( \gamma\right)=\frac{5\rho_{4}}{8}\cos6\theta\neq0,$$

$$\rho_{xy,1}\left( \beta\right)-\rho_{xy,1}\left( \gamma\right)=\rho_{13}-\rho_{16}\neq0,$$

$$\rho_{xy,3}\left( \beta\right)-\rho_{xy,3}\left( \gamma\right)=\rho_{14}-\rho_{17}\neq0,$$

$$\begin{aligned} \rho_{xy,5}\left( \beta\right)-\rho_{xy,5}\left( \gamma\right)=-\left( \rho_{13}+\rho_{14}-\rho_{16}-\rho_{17} \right)\neq0,\#\left( S.21 \right) \end{aligned}$$

but we have

$$\begin{aligned} \sum_{k=0} \rho_{xx,2k+1}\left( \beta\right)=\sum_{k=0} \rho_{xx,2k+1}\left( \gamma\right).\#\left( S.22 \right) \end{aligned}$$

In summary, it can be concluded that if the crystal axis is taken into account, the rule will change. Fig. 6(a) in the main text deviates from the two-vector model only at low temperatures, while they agree well at medium and high temperatures. This indicates that the influence of the crystal axis field cannot be neglected at low temperatures.

1. J. J. Sakurai/San Fu Tuan, Modern Quantum Mechanics (Revised Edition) (Addison Wesley Longman, Reading, MA, 1994).
2. Y. Miao, J. Sun, C. Gao, D. Xue, X.R. Wang, Anisotropic Galvanomagnetic Effects in Single Cubic Crystals: A Theory and Its Verification, Phys. Rev. Lett., 132, 206701 (2024)
